# Emergency Department-Based Screening and Linkage-to-Care for Hepatitis B, C, and D in a Referral Hospital in Barcelona, Spain: Reduction in Hepatitis C Prevalence After Five Years

**DOI:** 10.3390/v18090928

**Published:** 2026-08-24

**Authors:** Juan Carlos Ruiz-Cobo, Jordi Llaneras, Ariadna Rando-Segura, Ana Barreira-Díaz, Francisco Rodríguez-Frías, Judit Romero-Vico, Albert Blanco-Grau, Adriana Palom, Elena Vargas-Accarino, Roser Ferrer, Rafael Esteban, Mar Riveiro-Barciela, María Buti

**Affiliations:** 1 Medicine Department, Universitat Autònoma de Barcelona, 08193 Barcelona, Spain; juancarlosrcobo@gmail.com (J.C.R.-C.); mar.riveiro@vallhebron.cat (M.R.-B.); mariabutiferret@gmail.com (M.B.); 2 Liver Unit, Hospital Universitari Vall d’Hebron, 08035 Barcelona, Spain; 3 Centro de Investigación Biomédica en Red de Enfermedades Hepáticas y Digestivas (CIBEREHD), Instituto de Salud Carlos III, 28029 Madrid, Spain; 4 Vall d’Hebron Institut de Investigación, 08035 Barcelona, Spain; 5 Emergency Department, Hospital Universitari Vall d’Hebron, 08035 Barcelona, Spain; 6 Microbiology Department, Hospital Universitari Vall d’Hebron, 08035 Barcelona, Spain; 7 Biomedical Sciences Department, Universitat Internacional de Catalunya, 08017 Barcelona, Spain; 8 Nursery Department, Universitat de Barcelona, 08007 Barcelona, Spain; 9 Biochemistry Department, Hospital Universitari Vall d’Hebron, 08035 Barcelona, Spain

**Keywords:** emergency department, hepatitis B, hepatitis C, hepatitis D, screening, viral hepatitis

## Abstract

Background: Emergency Department (ED) viral hepatitis screening programs typically report short-term outcomes. We evaluated the medium-term impact of an ED-based screening for Hepatitis C (HCV), Hepatitis B (HBV), and Hepatitis D (HDV) viruses. Methods: Prospective opportunistic screening conducted between January 2020 and December 2024 at a referral hospital in Barcelona. Adults attending the ED were screened for HBV, HCV, and HDV. Positive cases were evaluated and linked to care when appropriate. To assess screening impact, HCV RNA and HBsAg prevalences were compared between 2020–2021 and 2023–2024 among participants aged ≤80 years. Results: Over five years, 39,532 individuals were screened: 220 (0.56%) had detectable HCV RNA, 234 (0.59%) HBsAg, and 4 HDV RNA. Of 110 patients with detectable HCV RNA eligible for linkage, 84 (76%) were treated and cured. Among 135 HBV-positive individuals eligible for linkage, 116 (86%) attended outpatient follow-up. HCV RNA prevalence significantly decreased (0.58% vs. 0.35%, *p* = 0.005), whereas HBsAg prevalence did not vary (0.7% vs. 0.58%, *p* = 0.224). Conclusions: Sustained ED-based screening enabled the identification and linkage to care of several individuals with HBV and HCV. This strategy contributed to a reduction in HCV prevalence among ED attendees and supports opportunistic ED screening in viral hepatitis elimination goals.

## 1. Introduction

Hepatitis B and C continue to pose major global health threats, causing approximately 1.1 million deaths yearly [1]. Although recent reports from the World Health Organization (WHO) indicate improvements in the diagnosis of these infections over the past decade, an estimated 90% of hepatitis B cases and 79% of hepatitis C cases remain undetected worldwide [2]. This widespread underdiagnosis limits access to appropriate antiviral therapy and contributes to persistent transmission. To address this, the WHO proposed a key operational shift in 2022 to eliminate hepatitis B and C as public health concerns by 2030: the simplification and integration of access to viral hepatitis testing [1].

However, a significant proportion of people with hepatitis B and C belong to vulnerable populations, such as migrants, people who inject drugs, and the homeless, who often have limited contact with conventional healthcare settings. Recent Spanish data have highlighted the disproportionately high prevalence of active HCV infection among socially vulnerable migrant populations, particularly among individuals of European origin and those with a history of injection drug use or alcohol misuse [3]. For many of them, the emergency department (ED) is the main or even only point of contact with the healthcare system. Thus, ED screening offers a valuable opportunity to diagnose and link to care [4,5,6,7,8].

Hepatitis C virus (HCV) prevalence is consistently higher in EDs than in the general population. A U.S. study comparing clinic-based and ED-based HCV screening programs reported markedly higher prevalence of both HCV antibodies (anti-HCV) (8% vs. 1%) and HCV RNA (3% vs. 0.08%) among ED attendees [9]. Other studies have reported an HCV prevalence of 0.2% to 3.9% [4,5,7,10]. These findings indicate the need for local data to achieve an efficient approach for viral hepatitis screening. Systematic reviews have confirmed that ED-based blood-borne virus screening is feasible across different healthcare systems and that it might identify both previously undiagnosed individuals and patients who have disengaged from follow-up. However, most published studies have focused on short-term implementation outcomes, and evidence regarding the long-term impact of sustained ED-based screening programs remains limited [10,11]. In a previous study [4], HCV RNA prevalence among patients attending our hospital’s ED was 0.7% after 2 years of opportunistic screening, nearly three times higher than the general Spanish population (0.22%) [12].

While HCV has been relatively well studied, there is less evidence regarding hepatitis B virus (HBV) prevalence in EDs. A systematic review of European ED-based screening programs reported HBsAg prevalences ranging from 0.2% to 0.9%, although relatively few studies included HBV testing [10]. More recently, a multicentre evaluation of routine opt-out HBV screening across seven UK emergency departments reported an HBsAg prevalence of 0.29%, confirming that HBV prevalence in European ED settings is consistently higher than that reported in the general population [13]. In our setting, HBV prevalence in the ED was 0.5%, also higher than the national estimate of 0.22% [4,12]. HDV infection, which occurs only in individuals with HBV infection, remains the most severe form of chronic viral hepatitis. Although it affects an estimated 2–5% of HBsAg-positive individuals, its true prevalence remains uncertain owing to geographical variation and limited systematic screening [14,15]. Given its association with worse clinical outcomes and the recent availability of effective antiviral therapy, reflex anti-HDV testing in HBsAg-positive individuals is increasingly recommended and may also improve knowledge of local HDV epidemiology [16,17,18,19].

An important ongoing controversy in viral hepatitis management is whether screening should be conducted universally [4,5,7,20], or limited to individuals with known risk factors [21]. Historically, most clinical guidelines recommended risk-based screening [22,23], which is considered more cost-effective. However, identifying individuals with risk factors can be challenging due to low public awareness, long latency period between infection and diagnosis, and the stigmatized nature of many risk factors, which may lead to under-reporting or denial. As a result, risk-based screening may miss a significant number of infected individuals. In our previous study [4], 60% of patients with HCV and 92% with HBV had no identifiable risk factors. Reflecting these limitations of risk-based approaches, recent recommendations from the Centers for Disease Control and Prevention (CDC) and the American Association for the Study of Liver Diseases (AASLD) now endorse one-time universal HBV and HCV screening for all adults [24,25,26].

That initial study, covering the first two years of ED hepatitis opportunistic screening, required oral patient consent, which was encouraged through iconographic material in patient-accessible areas of the ED. A cost-effectiveness analysis from the study demonstrated that viral hepatitis screening in our ED was economically justifiable [4]. Based on these results, this screening program was incorporated into the routine ED protocol.

Finally, most studies on ED-based viral hepatitis screening have been limited to short follow-up periods (<2 years), focusing on prevalence and early linkage to care [4,5,6,7,9,21]. In contrast, this study evaluates the medium-term (5-year) impact. Our aim was to assess the epidemiology of HBV, HCV, and HDV infection among ED attendees and to evaluate the impact of sustained ED-based screening and linkage to care on the epidemiology of viral hepatitis.

## 2. Materials and Methods

### 2.1. Study Design

This prospective, single-center study was carried out between January 2020 and December 2024 in the ED of an academic referral hospital in Barcelona, Spain. Hospital Universitari Vall d’Hebron covers a population of approximately 450,000 inhabitants. Starting in January 2020, individuals aged 18 years or older who attended the ED for any medical condition requiring a blood test were offered HBsAg and anti-HCV antibodies testing without additional sample collection. No formal exclusion criteria were applied, although screening could be omitted at the discretion of the attending ED physician when considered unlikely to provide clinical benefit. Positive samples underwent reflex testing for anti-HDV and HCV RNA respectively. All patients with positive results were evaluated, and linkage to care and antiviral treatment were offered when appropriate, based on an individual assessment by the coordinating hepatologist of the patient’s overall clinical condition and expected benefit from treatment.

However, a progressive decline in patient screening was observed from January 2022 onward, attributed to decreased healthcare provider motivation. To address this, the protocol was amended to waive the requirement for oral consent, and automated screening was implemented. Anti-HCV and HBsAg tests were automatically performed by a laboratory-based algorithm on ED blood samples from all individuals who had not undergone serological testing in the previous year. In addition, based on the low prevalence of HBV (<0.3%) and the limited number of patients eligible for HCV therapy (12 out of 78 with detectable HCV RNA) among individuals over 80 years of age in the previous study [4], this age group was excluded from the screening program at this stage. Notably, HCV RNA-positive patients considered ineligible for linkage to care during this period had a median age of 87 years (IQR 82.6–91.4).

To assess the impact of systematic viral hepatitis screening, the prevalence of HCV RNA and HBsAg was compared between the first (January 2020–December 2021) and last two years of screening (January 2023–December 2024). As individuals older than 80 years were excluded from screening after May 2023, only participants aged ≤80 years were included in this analysis. An additional analysis restricted to individuals aged 40–80 years was performed to evaluate the performance of the screening program according to the age range (40–90 years) proposed by a Spanish multidisciplinary consensus on ED-based HCV screening [27].

Each individual was planned to be screened once to avoid the unnecessary cost associated with repeated sampling and to determine viral hepatitis prevalence in the ED. All positive cases were evaluated by the coordinating physician before determining whether referral was necessary. Patients meeting treatment criteria were initiated on therapy during their first visit to minimize the risk of loss to follow-up.

### 2.2. Study Variables

Demographic characteristics, risk factors, and pertinent medical history were collected from all individuals who tested positive. Alanine aminotransferase (ALT) levels were considered within normal limits at values of 10–49 IU/L in men and 10–35 IU/L in women. Liver fibrosis was assessed using the FIB-4 score, with advanced fibrosis defined as a score > 3.25; positive cases were confirmed using transient elastography (FibroScan; Paris, France). Late presentation for medical care was defined by consensus as a serologic fibrosis score of ≥F3, transient elastography (FibroScan) reading of >9.5 kPa, FIB-4 > 3.25, or a liver biopsy result of ≥METAVIR stage F3, in patients without prior antiviral treatment [28].

Anti-HCV, HBsAg, and anti-HDV were determined by commercial tests: the Elecsys anti-HCV II assay (Roche Diagnostics; Rotkreuz, Switzerland), Elecsys HBsAg II assay (Roche Diagnostics), and LIASON XL anti-HDV assay (Diasorin; Saluggia, Italy). HCV RNA was determined using the Cobas HCV test on the Cobas 6800 system (Roche Diagnostics), with a lower limit of detection (LLD) of 9.61 IU/mL. HBsAg-positive patients were tested for HBV-DNA with the Cobas HBV test on the Cobas 6800 system (Roche Diagnostics) with an LLD of 2.4 IU/mL in serum. Anti-HDV patients were tested for HDV-RNA using an in-house 1-step quantitative RT-PCR using the WHO HDV-RNA international standard (PEI code number: 7657/12) with an LLD of 100 IU/mL.

For patients who were not considered for linkage to care, the reasons were recorded. Prospective data were collected, including linkage to care, initiation and completion of treatment. Successful linkage to care was defined as attendance at a hepatology outpatient clinic for initial clinical evaluation and management, including antiviral treatment when indicated. Cure of chronic HCV infection was defined as achieving a sustained virological response (SVR) 12 weeks after completing treatment.

### 2.3. Statistical Analysis

Variables with a normal distribution were compared using Student’s t test and expressed as mean ± standard deviation. Variables with a non-normal distribution were analyzed using the Mann–Whitney U test and expressed as median and interquartile range. Categorical variables were compared using the chi-square test or Fisher’s exact test when frequencies were less than 5% and expressed as frequency and percentage. Data were analyzed using StataCorp, 2015 (Stata Statistical Software, Release 14, College Station, TX, USA: StataCorp LP). As a sensitivity analysis, the association between screening period and HCV RNA positivity was assessed using Poisson regression with robust variance, adjusting for age and sex. Adjusted marginal prevalences were estimated for each screening period.

### 2.4. Ethical Considerations

All patient data were anonymized and encrypted.

## 3. Results

### 3.1. HBV, HCV, and HDV Prevalence in the ED

From January 2020 to December 2024, 45,003 ED samples were tested for anti-HCV and HBsAg. After excluding duplicates, 39,532 individuals were screened for viral hepatitis. The median age was 61 years (IQR 45–76), and 50.4% were male. Of these, 1405 (3.55%, 95% CI 3.38–3.74%) tested positive for anti-HCV, 220 (0.56%, 95% CI 0.49–0.64%) for HCV RNA, and 234 (0.59%, 95% CI 0.52–0.67%) for HBsAg. Anti-HDV was detected in 10 (4.27%, 95% CI 2.34–7.69%) of the 234 HBsAg-positive patients, and 4 of them tested positive for HDV RNA.

### 3.2. Characteristics of Anti-HCV-Positive Patients

Among the 1405 individuals testing positive for anti-HCV, the median age was 66 years (IQR 54–81), and 766 (54.5%) were male. Only 460 (32.7%) reported at least one risk factor, with the most common being a history of intravenous drug use (21%). Additionally, 189 (13.5%) were coinfected with human immunodeficiency virus (HIV). Previous anti-HCV positive testing had been performed in 849 (60.4%). Prior testing was more common among individuals born in Spain than among migrants (71.8% vs. 54.7%; *p* < 0.001). A comparison of the characteristics of new and previously known anti-HCV-positive cases is presented in Table 1.

### 3.3. Characteristics of HCV RNA-Positive Patients

Focusing on the 220 patients with detectable HCV RNA, their main characteristics are shown in Table 2. Of them, 118 (53.6%) had been tested previously and were aware of their infection. However, only 19 (16.2%) had started antiviral treatment at least once, with PEG-IFN being the most common prior therapy (52.9%). Risk factors were reported by 93 (42%). At the time of screening, 68 (30.9%, 95% CI 25–37%) met the criteria for late presentation to care, and 55 (25%, 95% CI 19–31%) had liver cirrhosis.

### 3.4. Linkage to Care of HCV Patients

Of the 220 patients with detectable HCV RNA, 110 (50%, 95% CI 43–57%) were not considered suitable for linkage to care. The reasons were: low life expectancy (n = 64, 58.2%), lack of contact information (n = 29, 26.4%), and death related to the medical condition that prompted the ED visit (n = 17, 15.4%).

Of the 110 patients eligible for linkage, 5 (4.5%) did not attend their visits. Consequently, among patients considered eligible for linkage to care, 105 (95.5%) were successfully linked to care and initiated treatment with pangenotypic DAAs. The median time from screening to treatment initiation was 72 days (IQR 39–167). Sustained virological response 12 weeks after end of treatment was assessed in 85 patients, with 98.8% (84/85) achieving SVR. Among the 20 individuals without SVR data, 14 had not yet reached their 12-week post-treatment visit at the time of writing, 3 died during follow-up from non-liver-related causes (2 from COVID-19), and 3 were lost to follow-up.

Of note, 31 of 55 patients with liver cirrhosis (56.4%) were linked to care and received treatment. Of these, 16 had previously been tested but were not linked. Twenty-four patients with cirrhosis had achieved SVR at the time of writing, two were lost to follow up, one died from COVID-19, and four are still awaiting the 12-week post-treatment visit.

**Figure 1:**
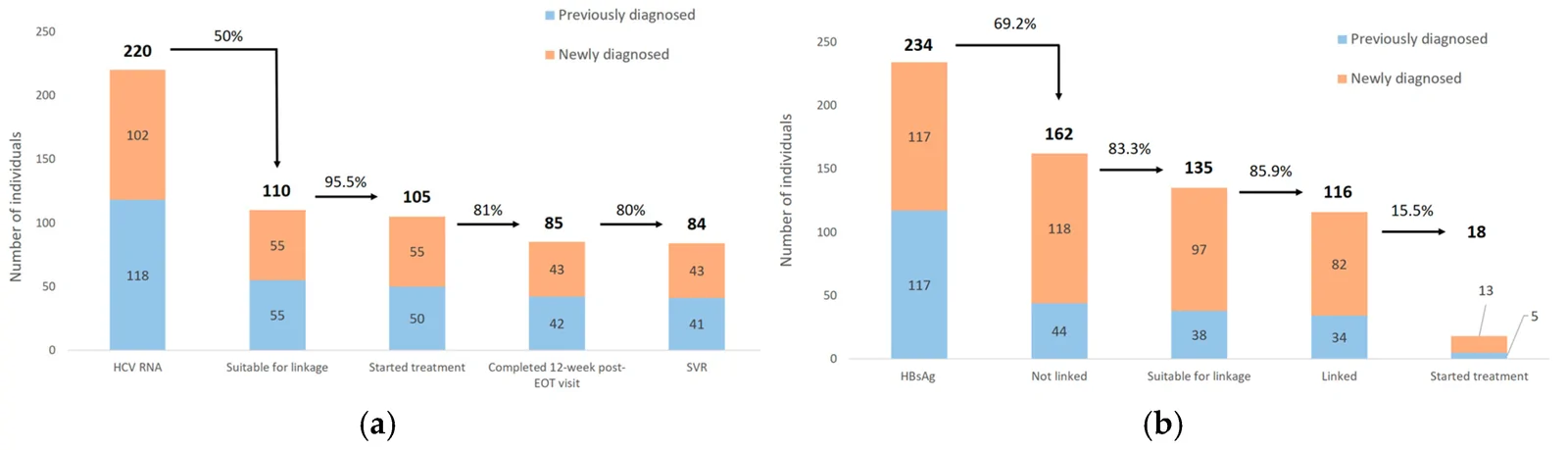
Linkage to care of HCV RNA-positive (**a**) and HBsAg-positive (**b**) individuals.

The linkage to care cascade for HCV RNA-positive patients is shown in Figure 1a.

### 3.5. Characteristics of HBsAg-Positive Patients

Baseline characteristics of the 234 individuals who tested positive for HBsAg are shown in Table 3. The median age was 57.5 (IQR 45–71.8) years, with most patients being male (64.5%) and White (65.8%). Only 56.4% were born in Spain, and 15% reported at least one risk factor. Half of them had previously been diagnosed with chronic HBV. A comparison between newly diagnosed patients and those who had already been tested is provided in Table 4. Among the 117 patients with a known HBV diagnosis, 45 (38.5%) were not receiving HBV-specific follow-up. In total, 30 patients (11.8%) had liver cirrhosis at the time of screening; of these, 10 were not linked to care and 5 had never been tested for HBsAg.

### 3.6. Linkage to Care of HBsAg-Positive Patients

The linkage to care cascade for HBsAg-positive individuals is shown in Figure 1b. Among the 162 patients without prior HBV care, 27 (16.6%) were not considered candidates for linkage. The reasons included low life expectancy (n = 11), death related to the medical condition that prompted the ED visit (n = 7), lack of contact information (n = 8), and refusal to be linked (n = 1). Among the 135 patients referred to the Hepatology outpatient clinic, 19 (14.1%) did not attend their scheduled visit. Ultimately, 18 patients were considered eligible for antiviral treatment, as they presented with HBeAg-positive (n = 3) or HBeAg-negative (n = 15) chronic hepatitis B.

### 3.7. Characteristics and Linkage to Care of Anti-HDV Positive Patients

Among the 10 patients who tested anti-HDV positive, 4 were newly diagnosed and 6 had a known history of HDV, of whom 2 were not linked. Among the 6 patients without prior HBV/HDV care, 5 were successfully linked to care. Only four individuals had detectable HDV RNA.

### 3.8. Impact of ED Viral Hepatitis Screening

**Figure 2:**
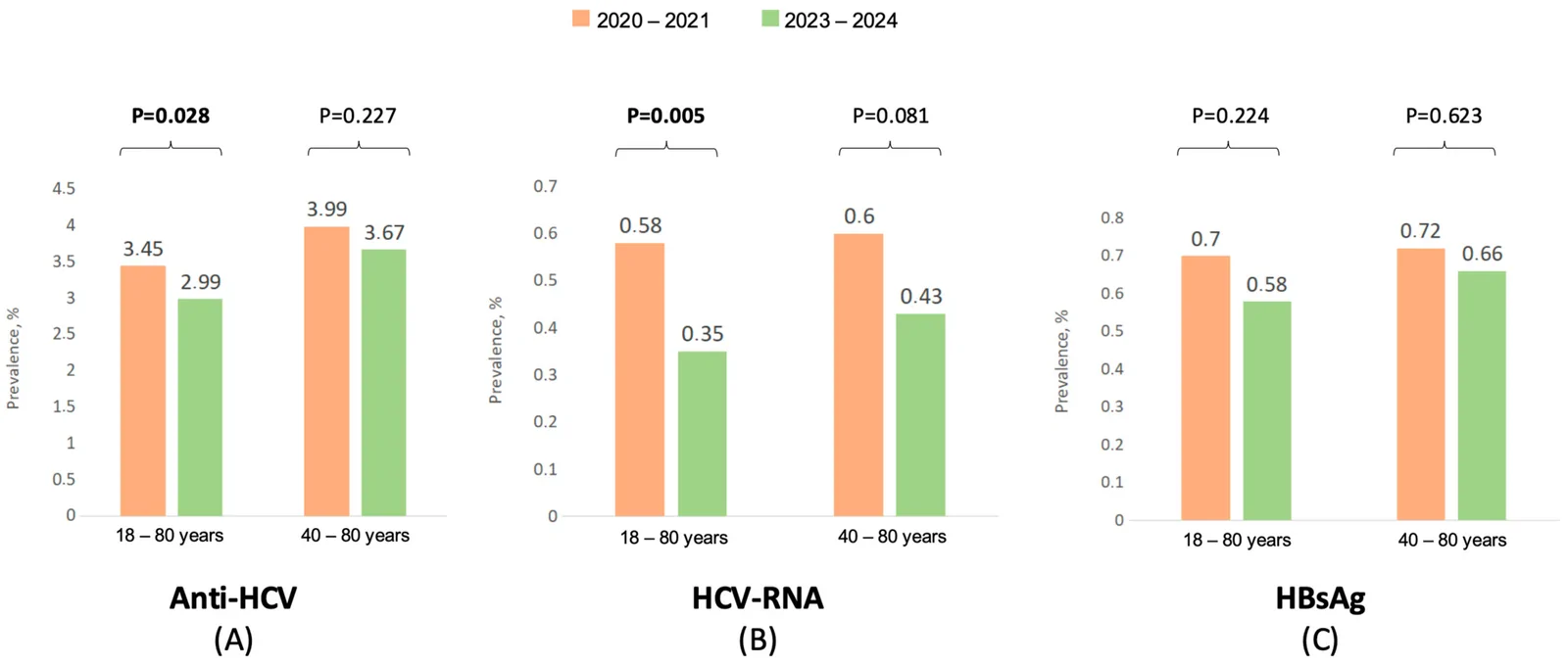
Prevalence of anti-HCV (**A**), HCV RNA (**B**), and HBsAg (**C**) during the first and second screening periods among patients aged 18–80 and 40–80 years.

During January 2020–December 2021 and January 2023–December 2024, 11,298 and 18,081 individuals aged 18–80 years were screened, respectively. Anti-HCV (a), HCV RNA (b), and HBsAg (c) prevalence rates in the first and second screening periods in patients aged 18 to 80 years and 40 to 80 years are shown in Figure 2. HCV RNA prevalence decreased significantly from 0.58% to 0.35% (*p* = 0.005). In a sensitivity analysis adjusting for age and sex, the reduction in HCV RNA prevalence persisted, with adjusted marginal prevalences of 0.54% in 2020–2021 and 0.34% in 2023–2024 (adjusted prevalence difference, −0.20 percentage points; 95% CI, −0.36 to −0.04; *p* = 0.010). This decline was also seen when analyzing HCV RNA prevalence among anti-HCV-positive individuals, which dropped from 16.67% to 11.67% (*p* = 0.034). No significant change was observed in HBsAg, which decreased from 0.70% to 0.58% (*p* = 0.224).

Differences in the baseline characteristics of individuals aged ≤80 years with active hepatitis C infection between the two screening periods are shown in Table 2. In the 2023–2024 period, there was a significant decrease in the proportion of Spanish-born patients (84.4% vs. 54%, *p* < 0.001), patients reporting risk factors (65.6% vs. 42.9%, *p* = 0.01), and patients with liver cirrhosis (28.1% vs. 19.4%, *p* = 0.002).

Baseline characteristics of HBsAg-positive individuals aged ≤80 years across the two screening periods are presented in Table 3. In 2023–2024, a smaller proportion of individuals had a previously known HBV infection compared to 2020–2021 (64.6% vs. 38.1%, *p* = 0.004). This difference may explain why a higher proportion of individuals in the earlier period were already linked to care at the time of screening (43% vs. 22.9%, *p* = 0.004). However, in the comparison of linkage to care after case evaluation, there were no significant differences between the 2 cohorts (87.3% vs. 81%, *p* = 0.303).

## 4. Discussion

To our knowledge, this is the longest study to date evaluating hepatitis B, C and D screening in the ED. The sustained implementation of a viral hepatitis screening and linkage-to-care program over a five-year period was associated with a meaningful reduction in the prevalence of HCV infection among patients aged ≤80 years from 0.58% to 0.35%. In contrast, no significant differences were observed in HBsAg prevalence. The prevalence of HDV coinfection was low (4.27% of HBsAg-positive) but consistent with prior evidence [15]. However, their identification remains clinically relevant given the severity of HDV infection and the recent availability of effective antiviral therapy.

Our findings highlight the importance of opportunistic screening in capturing infections missed by risk-based strategies. Previous reports found that 86% of anti-HCV-positive patients have identifiable risk factors for viral hepatitis [29]. In contrast, less than half of patients with detectable HCV RNA and only 15% of HBsAg-positive patients reported recognised risk factors, underscoring the limitations of risk-based screening. Differences in the proportion of patients reporting recognised risk factors across studies may reflect variations in the populations served by individual EDs, language and cultural barriers, and the reluctance of some individuals to disclose stigmatised exposures such as injecting drug use. Previously untested individuals were less likely to report risk exposures but had a higher prevalence of detectable HCV RNA, emphasising the limitations of current screening approaches and supporting broader testing policies. Moreover, when both risk factors and elevated transaminase levels were considered, only 62.7% of individuals with detectable HCV RNA and 28.6% of those positive for HBsAg would have met current screening criteria. Consistent with AASLD and CDC recommendations, our results support universal one-time HBV and HCV screening in adults [24,25,26].

A quarter of HCV RNA-positive and 12.8% of HBsAg-positive patients already had cirrhosis at screening, including individuals who had never previously been tested. Although the proportion of cirrhosis decreased significantly among HCV RNA-positive patients between 2020–2021 and 2023–2024 (28.1% vs. 19.4%, *p* = 0.002), this difference was not statistically significant among HBsAg-positive patients (16.5% vs. 9.5%, *p* = 0.119). Nevertheless, a significant decline in late presentation to care among HBV-infected individuals was observed. This suggests that ED-based programs may contribute to earlier diagnosis and engagement in care, potentially preventing progression and improving long-term outcomes.

Anti-HCV-positive individuals who had never been tested were older (74 vs. 60 years, *p* < 0.001), more likely to be women (54% vs. 39.9%, *p* < 0.001), and more frequently migrants (30.3% vs. 19.5%, *p* < 0.001). This was not observed among HBsAg-positive patients. These findings may reflect inequities in access to HCV testing related to factors such as the historical exclusion of women from the workforce (and thus from occupational health screenings), reduced clinical suspicion in older adults, and structural barriers to healthcare access among migrant populations. These findings reinforce the rationale for universal opportunistic screening, particularly in the ED, as risk-based HCV screening may disproportionately miss these populations.

Social vulnerability among ED attendees remains a key consideration. More than half of viraemic HCV patients and one in five HBsAg-positive patients had previously been diagnosed but were not engaged in care. While earlier reports suggested that ED screening identified cases without improving treatment rates, our program achieved high linkage outcomes, with 95.5% of HCV RNA-positive and 85.9% of HBV-positive patients eligible for follow-up successfully entering care. Linkage-to-care rates reported in previous ED-based screening programs have varied widely, ranging from 12.9% to 100% [10,11]. Nevertheless, our linkage-to-care rates were higher than those reported in many previous HCV or HBV screening programs. This may be explained by the integration of the program within a universal healthcare system, the availability of a coordinating hepatologist responsible for patient contact and follow-up, and a predefined referral pathway to the Liver Unit. This demonstrates the value of EDs not only as diagnostic sites but also as critical gateways into the care cascade.

From an implementation perspective, automation proved essential. Manual, consent-based screening was effective initially but declined over time. The transition to automated universal testing ensured sustainability, minimized missed opportunities, and increased efficiency by preventing unnecessary retesting. Automation also enabled flexible adjustment of eligibility criteria in response to evolving epidemiology. These findings demonstrate that automation is essential for the long-term sustainability of ED-based viral hepatitis screening programs, overcoming the limitations of manual screening and enabling flexible adaptation to changing epidemiology.

This study has some limitations. First, as this was a single-centre study conducted within a universal healthcare system, our findings may not be directly generalizable to other healthcare settings. Although the reduction in HCV RNA prevalence persisted after adjustment for age and sex, country of birth and risk-factor information were not systematically available for the entire screened population and therefore could not be included in the sensitivity analysis. Moreover, as individuals attending the ED do not constitute a closed population, reductions in HCV prevalence may partially reflect concurrent microelimination strategies such as retrospective database search for HCV RNA-positive cases and prospective screening for viral hepatitis in primary care centers [30,31]. Indeed, reductions in active HCV infection have been reported in other Spanish cohorts. A recent study showed a decline in active HCV prevalence from 7.2% to 3.4% (*p* = 0.04) among people experiencing homelessness in Madrid between 2019 and 2023, which was attributed to active screening and treatment strategies, supporting the broader impact of HCV elimination efforts nationwide [32]. Occult HBV infection was not assessed, and therefore the reported HBV prevalence reflects HBsAg-positive infection and may underestimate the overall burden of HBV infection in the ED population. Additionally, before automation, ED clinicians initiated testing, potentially introducing selection bias toward higher-risk groups. Therefore, temporal changes in HCV prevalence should be interpreted with caution, as they may reflect changes in the screened population, the screening strategy, and concurrent HCV microelimination interventions. Nevertheless, this reflects real-world program evolution and underscores the benefits of automation.

In conclusion, after five years of ED-based screening with high linkage-to-care rates for HBV and HCV, a significant reduction in HCV RNA prevalence was observed among ED attendees. While HBV prevalence remained unchanged, late presentation decreased. These findings support the role of ED-based screening as a valuable component of viral hepatitis elimination strategies.

## Figures and Tables

**Table 1:** Characteristics of anti-HCV-positive patients by prior HCV screening history.

	New Diagnosis (n = 556)	Previously Tested (n = 849)	*p* Value
Age (years), Md (IQR)	74 (57–87)	60 (53–75)	**<0.001**
Male sex, n (%)	256 (46)	510 (60.1)	**<0.001**
Born in Spain, n (%)	202/290 (69.7)	410/503 (81.5)	**<0.001**
Detectable HCV RNA, n (%)	110 (19.8)	110 (13)	**<0.001**
Risk factors, n (%)	86 (15.5)	374 (44)	**<0.001**
ALT (IU/L), Md (IQR)	20 (13–32)	21 (14–35)	0.094
Elevated ALT, n (%)	97 (17.4)	145 (17.1)	0.847
Elevated ALT or risk factors, n (%)	158 (28.4)	448 (52.8)	**<0.001**
Liver cirrhosis, n (%)	29 (5.2)	247 (29.1)	**<0.001**

Data are shown as number of patients (%) or median (IQR). ALT, Alanine aminotransferase.

**Table 2:** Baseline characteristics of HCV RNA-positive patients screened in 2020–2021 vs. 2023–2024 (excluding patients > 80 years old).

	Total (n = 220) *	2020–2021 (n = 64)	2023–2024 (n = 63)	*p* Value
Age (years), Md (IQR)	65.5 (52–87)	54 (43–67)	53 (47.5–60)	0.956
Male sex, n (%)	116 (52.7)	43 (67.2)	47 (74.6)	0.368
Born in Spain, n (%)	178 (81.3)	58 (84.4)	34 (54)	**<0.001**
Race/Ethnicity, n (%)				0.121
White	199 (90.5)	59 (92.2)	50 (79.4)	
Latino	9 (4.1)	2 (3.1)	5 (7.9)	
Asian	3 (1.4)	0	3 (4.8)	
Black	1 (0.5)	1 (1.6)	0	
Middle Eastern/North African	8 (3.6)	2 (3.1)	5 (7.9)	
Risk factors, n (%)	93 (42.3)	42 (65.6)	27 (42.9)	**0.01** **0**
Previously tested for anti-HCV, n (%)	118 (53.6)	34 (53.1)	37 (60.7)	0.329
ALT (IU/L), Md (IQR)	31 (19–60.3)	48 (19–97)	45 (27.5–75.5)	0.534
Elevated ALT, n (%)	85 (38.6)	31 (48.4)	30 (47.6)	0.926
Elevated ALT or risk factors, n (%)	138 (62.7)	52 (81.3)	43 (68.3)	0.092
FIB-4, Md (IQR)	2.6 (1.6–4.8)	2.3 (1.5–4.7)	2.1 (1.1–3.1)	**0.045**
APRI, Md (IQR)	0.7 (0.4–1.3)	0.8 (0.4–1.7)	0.7 (0.4–1.1)	0.074
Late presentation to care, n (%)	68 (30.9)	21 (32.8)	19 (30.2)	0.448
Liver cirrhosis, n (%)	55 (25)	18 (28.1)	12 (19.4)	**0.002**

Data are shown as number of patients (%) or median (IQR). *p* values were obtained from univariate analyses with Bonferroni correction for multiple comparisons. ALT, Alanine aminotransferase; APRI, AST-to-Platelet Ratio Index; FIB-4, Fibrosis-4 Index; IQR, Interquartile Range. * This column includes all patients identified during the entire screening period (2020–2024).

**Table 3:** Baseline characteristics of HBsAg-positive patients screened in 2020–2021 vs. 2023–2024 (excluding patients > 80 years old).

	Total (n = 234) *	2020–2021 (n = 79)	2023–2024 (n = 105)	*p* Value
Age (years), Md (IQR)	57.5 (45–71.8)	58 (45.5–69)	52 (45–67)	0.466
Male sex, n (%)	151 (64.5)	58 (73.4)	66 (62.9)	0.087
Born in Spain, n (%)	132 (56.4)	46 (58.2)	56 (53.3)	0.351
Race/Ethnicity, n (%)				0.175
White	154 (65.8)	51 (64.6)	66 (62.9)	
Latino	17 (7.3)	6 (7.6)	9 (8.6)	
Asian	16 (6.8)	2 (2.5)	12 (11.4)	
Black	24 (10.3)	10 (12.7)	10 (9.5)	
Middle Eastern/North African	23 (9.8)	10 (12.7)	8 (7.6)	
Risk factors, n (%)	35 (15)	8 (10.1)	19 (18.1)	0.131
Previously tested for HBsAg, n (%)	117 (50)	51 (64.6)	40 (38.1)	**0.004**
ALT, IU/L	23 (15–36)	26 (15.5–37)	22 (16–34)	0.194
Elevated ALT, n (%)	40 (17.1)	13 (16.5)	19 (18.1)	0.771
Elevated ALT or risk factors, n (%)	67 (28.6)	21 (26.6)	32 (30.5)	0.564
FIB-4, Md (IQR)	1.5 (0.9–2.5)	1.6 (1.0–2.5)	1.2 (0.8–2.0)	0.053
APRI, Md (IQR)	0.4 (0.3–0.6)	0.4 (0.3–0.6)	0.3 (0.2–0.5)	**0.025**
Late presentation to care, n (%)	37 (15.8)	18 (22.8)	10 (9.5)	**0.012**
Cirrhosis, n (%)	30 (12.8)	13 (16.5)	10 (9.5)	0.119
Anti-HDV, n (%)	10 (4.3)	2 (2.7)	5 (4.8)	0.701
Linked to care before, n (%)	72 (30.8)	34 (43)	24 (22.9)	**0.004**
Linked to care after, n (%)	185 (79.1)	69 (87.3)	85 (81)	0.303

Data are shown as number of patients (%) or median (IQR). *p* values were obtained from univariate analyses with Bonferroni correction for multiple comparisons. ALT, Alanine aminotransferase; APRI, AST-to-Platelet Ratio Index; FIB-4, Fibrosis-4 Index; IQR, Interquartile Range. * This column includes all patients identified during the entire screening period (2020–2024).

**Table 4:** Characteristics of HBsAg-positive patients by prior HBsAg screening history.

Characteristic	New Diagnosis (n = 117)	Previously Tested (n = 117)	*p* Value
Age (years), Md (IQR)	56 (45–71)	58 (48–72)	0.266
Male sex, n (%)	81 (69.2)	70 (59.8)	0.133
Born in Spain, n (%)	60 (51.3)	72 (61.5)	0.114
Risk factors, n (%)	12 (10.3)	23 (19.7)	**0.044**
ALT (IU/L), Md (IQR)	22 (15–35.3)	24 (15–36)	0.636
Elevated ALT, n (%)	20 (17.1)	20 (17.1)	1.000
Elevated ALT or risk factors, n (%)	29 (24.8)	38 (32.5)	0.193
Positive anti-HDV, n (%)	4 (3.4)	6 (5.1)	0.748
HBeAg positive, n (%)	6 (5.1)	2 (1.7)	0.281
Late presentation to care, n (%)	7 (6)	30 (25.6)	**<0.001**
Liver cirrhosis, n (%)	5 (4.3)	25 (21.4)	**<0.001**

Data are shown as number of patients (%) or median (IQR). ALT, Alanine aminotransferase.

## Data Availability

The data supporting the findings of this study are available upon reasonable request to the corresponding author.

## Abbreviations

The following abbreviations are used in this manuscript:

AASLD	American Association for the Study of Liver Diseases
ALT	Alanine aminotransferase
anti-HCV	Antibody to hepatitis C virus
anti-HDV	Antibody to hepatitis D virus
APRI	AST-to-Platelet Ratio Index
CDC	Centers for Disease Control and Prevention
DAAs	Direct-acting antivirals
ED	Emergency Department
FIB-4	Fibrosis-4 Index
HBV	Hepatitis B virus
HCV	Hepatitis C virus
HDV	Hepatitis D virus
HIV	Human immunodeficiency virus
LLD	Lower limit of detection
SVR	Sustained virological response
WHO	World Health Organization
